# Safety and Efficacy of Triple Therapeutic Targets with Rivaroxaban after Acute Myocardial Infarction Complicated by Left Ventricular Thrombi in a Case of Nonvalvular Atrial Fibrillation

**DOI:** 10.1155/2018/6503435

**Published:** 2018-03-05

**Authors:** Francesco Summaria, Gregory A. Sgueglia, Fabrizio D'Errico, Antonella De Santis, Fabiana Piccioni, Gaetano Gioffrè, Achille Gaspardone

**Affiliations:** Department of Cardiology, San Eugenio Hospital, Rome, Italy

## Abstract

We present the complex case of a high-risk patient with nonvalvular atrial fibrillation, who experienced a non-ST elevation myocardial infarction complicated by left ventricular (LV) thrombi and underwent percutaneous coronary intervention with drug-eluting stent implantation. The patient was initially treated with short-term triple therapy including aspirin, clopidogrel, and rivaroxaban 15 mg/die. Following aspirin dropping one month after discharge, the patient continued on dual therapy with clopidogrel and rivaroxaban, and a clinical and imaging follow-up at 6 and 12 months confirmed the LV thrombi resolution, with no thromboembolic episodes and a good safety profile.

## 1. Introduction

Among acute coronary syndrome (ACS) patients undergoing percutaneous coronary intervention (PCI), approximately 5% to 22% have concomitant atrial fibrillation (AF) [1]. Among ACSs, AF is 2-fold more frequent during non-ST elevation myocardial infarction (NSTEMI) than during STEMI [2]. Despite the overlap in the occurrence of these syndromes, the pharmacotherapies used to manage AF and ACSs differ.

Left ventricular (LV) thrombi often represent an unexpected echocardiographic finding [3], accurately detected by cardiovascular magnetic resonance (CMR) [4]. Although the occurrence of this complication has declined significantly since the advent of primary PCI, its incidence is not negligible, ranging between 5% and 17% [5, 6].

According to current guidelines, despite an increased rate of bleeding complications, warfarin is considered the anticoagulant of choice to prevent thromboembolic sequelae. As an advantageous alternative, the novel oral anticoagulants (NOACs) have been implemented in the setting of nonvalvular AF (NVAF) [7–9]. Yet, the evidence about their role in combination with antiplatelet agents in the management of LV thrombi for ACS patients undergoing PCI is poor, and mostly limited to case reports. Hence, their efficacy can be only extrapolated [10–13]. At present, two randomized trials (PIONEER AF-PCI and RE-Dual PCI) [14, 15] have demonstrated that the use of NOACs plus a P2Y12 inhibitor was associated with a lower rate of clinically significant bleeding, compared to VKAs plus dual antiplatelet therapy, thus ensuring a dual target of ischemic and thromboembolic protection against coronary and cerebrovascular events.

## 2. Case Report

**Figure 1:**
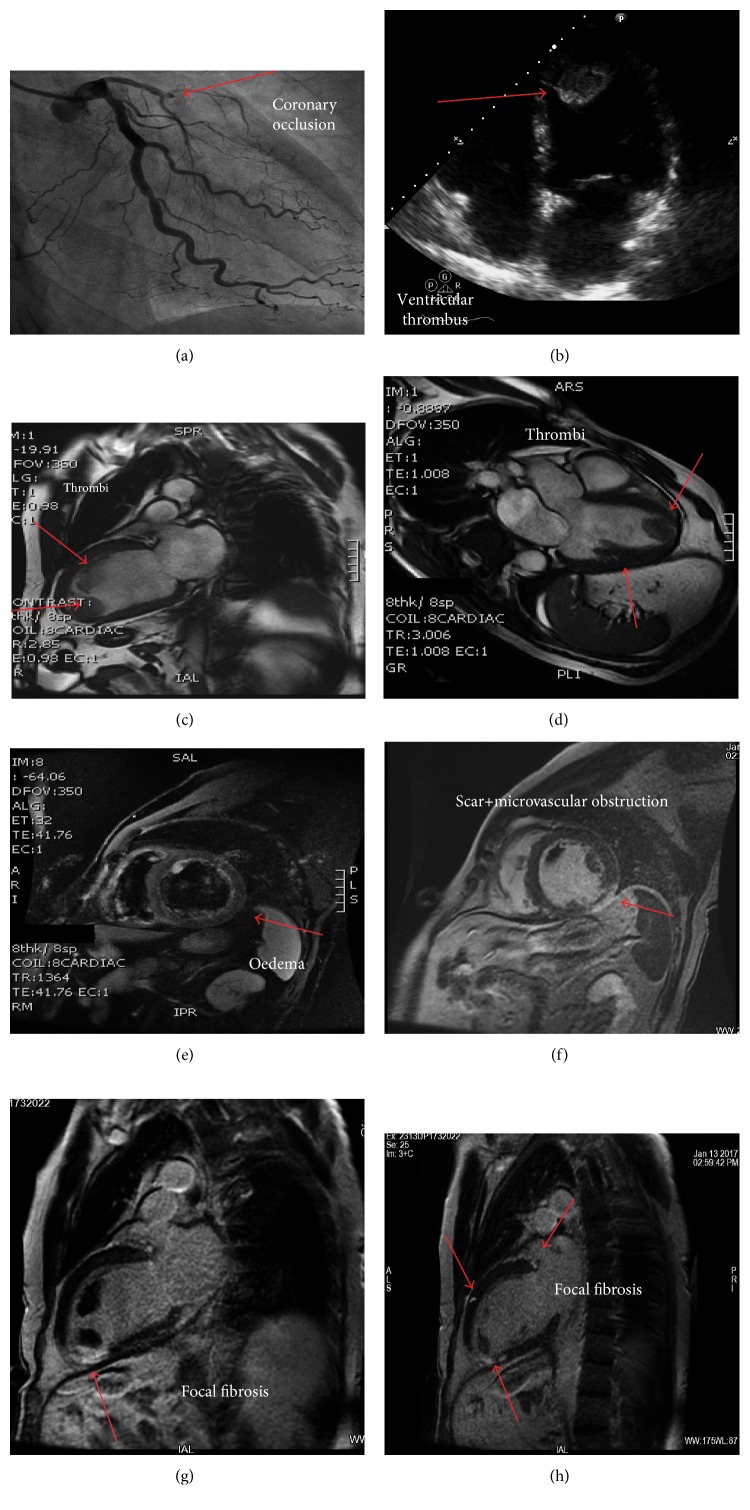
(a) Coronary angiography showing the occlusion of the left descending artery in the proximal segment (arrow); (b) transthoracic 2-D echocardiography showing an apical ventricular thrombus; (c, d) cardiac magnetic resonance (CMR) unveiling the presence of two large left ventricular thrombi in the apex and along the anterior wall; (e) CMR TIR-T2 sequences showing myocardial edema involving the anterior wall of the left ventricle; (f, g) delay enhancement revealing scar, microvascular obstruction, and fibrosis (arrows); (h) CMR imaging: three hyperenhancement focal areas of fibrosis.

We report the case of a 66-year-old woman, hypertensive, and smoker, referred to our Emergency Department for prolonged chest pain. Due to permanent NVAF, she was on warfarin and rate-control therapeutic strategy. At admission, electrocardiogram showed AF and a ST segment depression of 2 mm in V5-V6. The creatine kinase-MB was of 10 and high-sensitivity troponin I was of 6.0 ng/mL, with normal hemoglobin level and INR of 1.9. At baseline, a combined thromboembolic and bleeding scoring system evaluation was performed, resulting in a CHA_2_DS_2_-VASc of 4 and HAS-BLED of 3 [16–18]. A NSTEMI diagnosis was made, and an antiplatelet therapy with aspirin 300 mg, clopidogrel 600 mg loading dose, and intravenous unfractionated heparin 5000 IU was administered, while an early invasive strategy with a transradial approach was planned. The coronary angiography showed a normal right coronary artery and the occlusion of the left descending artery in the proximal segment (Figure 1(a)). An IVUS-guided PCI was performed, with the implantation of two overlapped last-generation DES (Xience Alpine, Abbott), 2.75 × 28 mm and 3.0 × 28 mm, respectively, both expanded up to 16 atmospheres. In order to optimize expansion and avoid malapposition, the stents were overexpanded, with noncompliant balloons of 3.5 × 15 mm (NC Quantum, Boston), up to 20 atmospheres, according to IVUS-guided vessel sizing.

Considering the clinical setting (NSTEMI + AF) and the procedure (PCI with DES implantation), the antiplatelet therapy with aspirin 100 mg/day plus clopidogrel 75 mg/day was continued and anticoagulation with rivaroxaban 15 mg/day was started immediately after PCI. The transthoracic 2-D echocardiography revealed a hypokinesia of the apex and anterior wall with moderate reduction of the ejection fraction. Two mobile masses were found in the apex and in the anterior wall of the left ventricle, respectively (Figure 1(b)). A CMR was performed at day three, confirming apex and anterior wall hypokinesia and 40% ejection fraction. CMR unveiled the presence of two LV thrombi in the apex and along the anterior wall, respectively (Figures 1(c) and 1(d)). TIR-T2 sequences showed myocardial oedema (Figure 1(e)) involving the LV anterior wall; delay enhancement (Figures 1(f) and 1(g)) revealed a thickness fibrosis in the same segment, with a hypoenhanced internal area suggesting microvascular obstruction. Three hyperenhancement focal areas were detected (Figure 1(h)): a transmural area at inferior apical septum, just beneath one thrombus, and two subepicardial areas, at anterior basal septum and at midanterior wall, respectively.

The patient was discharged 7 days after admission. A clinical and cardiovascular imaging follow-up was planned at 1 month, 6 months, and 1 year. According to current guidelines and expert consensus knowledge [19, 20], the antithrombotic/anticoagulant therapy was managed as follows: at 1 month, the thromboembolic and bleeding risk scores (CHA_2_DS_2_-VASc of 4 and HAS-BLED of 3) were rechecked, the antiplatelet therapy with clopidogrel together with the anticoagulant therapy with rivaroxaban 15 mg/day was confirmed, and aspirin was stopped considering the high bleeding risk. At 6 months, no ischemic thromboembolic or bleeding events were reported. The echo and CMR imaging confirmed the resolution of the LV thrombi. Despite the fact that MRI are not necessary to confirm the thrombi resolution documented by echocardiogram, in our institution CMR facility together with an internal protocol in the case of a newer therapeutic strategy led us to perform this modality of cardiac imaging for speculative purpose. The dual therapy with clopidogrel plus rivaroxaban was confirmed [14].

At 1 year, a clinical follow-up confirmed the good clinical results: the patient was asymptomatic, and neither ischemic coronary events nor thromboembolic or bleeding events were reported. After 1 year, considering the CHA_2_DS_2_-VASc score, the high rate of recurrence of events after NSTEMI, the use of DES, the absence of bleeding events during previously dual antiplatelet therapy, a treatment with rivaroxaban plus aspirin was confirmed.

## 3. Discussion

The optimal antithrombotic strategy for patients with NVAF and ACS undergoing PCI is still controversial, and the role of NOACs in this setting is poorly documented [10–14].

The guidelines of ACS and AF report how to manage the antiplatelet and anticoagulant therapies in overlapped situations, but, frequently, the indication and level of evidence are strong only when the conditions are considered alone [19, 20]. Many different combinations have been proposed based on the use of different agents and for varying duration, but as the final target must balance the risk of bleeding and ischemic events in each patient, it is difficult to make a definitive decision especially when other potential thromboembolic complications (e.g., LV thrombi) occur. NOACs are at least as effective as warfarin in terms of thromboembolic complications, but safer in terms of bleeding complications [8, 9]. As for the dose, in the dual or triple therapy, guidelines recommend the lower available dose of NOACs [19, 20]. Based on the recent results of the PIONEER AF-PCI trial [14], the more effective and safer ratio is clopidogrel 75 mg plus rivaroxaban 15 mg, or either clopidogrel or ticagrelor plus dabigatran at 110 mg or 150 mg (RE-DUAL PCI) [21]. In contrast with the currently available data in which the evidence of the efficacy and safety of NOACs in patients with ACS and AF undergoing PCI is limited to a low HAS-BLED score, our patient had a high bleeding risk; therefore, despite this acute setting, a triple therapy was used for 4 weeks only, followed by dual therapy with clopidogrel plus rivaroxaban 15 mg/die for 12 months [14].

The use of aspirin has been reconsidered in the subgroup of patients at high risk of bleeding: for 6 months only in case of low bleeding risk, while for one month in case of high bleeding risk [19, 20]. However, given the results of PIONEER and RE-Dual PCI, it is likely that aspirin will be definitively abandoned.

Periprocedural and technical cautions are crucial to minimize either the risk of access site bleeding or the risk of stent thrombosis and recurrence of coronary events. The transradial approach is mandatory also because it shortens the time to restart oral anticoagulation. In our case, the use of the latest-generation fluoro-passivated everolimus-eluting stent, as recently documented [22], lowers the rate of stent thrombosis and thrombotic complications. Intracoronary imaging with an IVUS-guided stent implantation reduces vessel wall malapposition and, consequently, stent thrombosis, thus improving a tailored pharmacological management.

Our combined interventional and pharmacological strategy aimed at balancing the ischemic and bleeding risk was also based on the use of the lower dose of NOAC tested for stroke prevention, and clopidogrel instead of the more potent ticagrelor and prasugrel, as suggested by guidelines and expert consensus [19, 20].

Although coronary DES significantly reduced the recurrences during the follow-up, the likelihood of a new event is not negligible. In this context, the use of NOACS seems to be a safe and effective option when the patient complexity and frailty due to higher combined thromboembolic and bleeding risk require to achieve multiple therapeutic targets.
